# Changes in copeptin levels before and 3 months after transsphenoidal surgery according to the presence of postoperative central diabetes insipidus

**DOI:** 10.1038/s41598-021-95500-x

**Published:** 2021-08-26

**Authors:** Yoo Hyung Kim, Yong Hwy Kim, Young Soo Je, Kyoung Ryul Lee, Hwan Sub Lim, Jung Hee Kim

**Affiliations:** 1 Department of Internal Medicine, Seoul National University Hospital, Seoul National University College of Medicine, Seoul, 03080 Korea; 2 Department of Neurosurgery, Seoul National University Hospital, Seoul National University College of Medicine, Daehak-ro, Jongno-gu, Seoul, 10103080 Korea; 3 Pituitary Center, Seoul National University Hospital, Seoul, 03080 Korea; 4 Seoul Clinical Laboratories, Heungdeok IT Valley 25F, HeungDeok 1-ro 13, Giheung Gu, YongIn City, 16954 Gyeonggido Korea

**Keywords:** Pituitary diseases, Endocrine cancer, Neuroendocrine diseases

## Abstract

Copeptin levels reflect arginine vasopressin (AVP) release from the hypothalamus. Pituitary surgery often impairs AVP release and results in central diabetes insipidus (CDI). Here, we aimed to investigate how serum copeptin level changes 3 months after pituitary surgery and whether it has a diagnostic value for postoperative permanent CDI. Consecutive patients who underwent endoscopic transsphenoidal surgery at a single tertiary hospital were recruited. Serum copeptin levels were measured preoperatively and 3 months postoperatively. Among 88 patients, transient and permanent CDI occurred in 17 (19.3%) and 23 (26.1%), respectively. Three-month postoperative copeptin levels significantly declined from preoperative levels in permanent CDI group (*P* < 0.001, percentage difference =  − 42.2%) and also in the transient CDI group (*P* = 0.002, − 27.2%). Three months postoperative copeptin level < 1.9 pmol/L under normal serum sodium levels was the optimal cutoff value for diagnosing permanent CDI with an accuracy of 81.8%, while 3-month postoperative copeptin level ≥ 3.5 pmol/L excluded the CDI with a negative predictive value of 100%. Conclusively, 3 months postoperative copeptin levels significantly decreased from preoperative levels in the transient CDI group as well as the permanent CDI group. Three-month postoperative copeptin levels ≥ 3.5 pmol/L under normal serum sodium levels may be diagnostic for excluding postoperative CDI.

## Introduction

Arginine vasopressin (AVP) is synthesized in magnocellular neurons of the supraoptic and paraventricular nuclei of the hypothalamus and stored in the posterior pituitary gland. AVP is the primary regulator of osmolar homeostasis through water reabsorption of renal tubules in response to the plasma osmolality and circulating volume. Copeptin is cleaved from the C-terminal proportion of pre-pro-vasopressin. Although the physiologic role of copeptin is still inconclusive, a few studies have reported that it acts as a chaperone protein and involves the accurate folding of the AVP precursor^1^. Copeptin is also secreted into the bloodstream together with AVP upon osmotic stimulation but has a longer half-life and higher stability than AVP^2–4^.

Central diabetes insipidus (CDI) is a polydipsia–polyuria syndrome due to the altered synthesis and secretion of AVP. CDI often occurs after the surgical resection of pituitary tumors (16–34%), which accounts for electrolyte imbalance and more extended hospitalization^5–8^. Transient postoperative CDI related to the dysfunction of AVP-secreting neurons occurs 24–48 h after surgery and resolves within 10 days^8^. Permanent postoperative CDI rarely occurs since at least 80–90% of AVP secreting neurons are irreversibly damaged^9^. Several risk factors, including tumor pathology (i.e., craniopharyngioma), tumor size, the extent of surgical resection, previous surgery, visual field defect, and intraoperative cerebrospinal fluid leakage, have been identified for postoperative CDI^8,10–13^. We also demonstrated that the cephalocaudal tumor diameter in nonfunctioning pituitary adenoma was a predictor of postoperative CDI^14^.

Unrecognized postoperative CDI often results in hypernatremia, hyperosmolarity, and progressive symptoms and signs, including lethargy, irritability, and even seizures^15^. Unfortunately, the diagnosis of postoperative CDI is still contingent on the symptom of polydipsia and polyuria because of the lack of rapid and effective diagnostics. Although direct AVP measurement in symptomatic patients is crucial in diagnosing CDI, it is not commonly used in clinical practice owing to the technical limitations and low accuracy of the AVP commercial assay^6,16^. Instead, the indirect water deprivation test or hypertonic saline test is widely used in diagnosing CDI. Recently, reliable commercial assays of copeptin are available. Unlike AVP, copeptin is stable at room temperature for 7 days, requiring only a small sample volume (50 µL of serum or plasma), and has a short turnaround time (0.5–2.5 h)^2,17,18^. Moreover, several researchers have suggested that copeptin is useful for the differential diagnosis of polydipsia–polyuria syndrome^19,20^. Fenske et al. has exhibited that baseline copeptin values < 2.6 pmol/L indicated complete CI^19^. Others have also demonstrated that hypertonic saline-stimulated copeptin with ≤ 4.9 pmol/L and arginine-stimulated copeptin with ≤ 3.8 pmol/L^5,20^ had a high accuracy of more than 95%. Previous studies suggested that low copeptin levels at the immediate postoperative time predicted the permanent postoperative CDI since the postsurgical stress stimulates copeptin secretion^21–23^. However, the diagnostic value of 3-month postoperative copeptin levels for postoperative CDI and the change of 3-month postoperative copeptin level from the preoperative level have not been studied.

Here, we first compared the change of serum copeptin levels before and 3 months after transsphenoidal surgery according to the presence of CDI. Furthermore, we aimed to show whether 3-month postoperative copeptin levels have a diagnostic value for postoperative CDI and suggest the cutoff value of copeptin levels for diagnosing CDI. We also assessed the predictive risk factors for permanent CDI after transsphenoidal surgery.

## Results

### Baseline characteristics according to the presence of postoperative CDI

Among a total of 88 patients, permanent CDI was observed in 23 (26.1%) patients, while transient DI and non-CDI were noted in 17 patients (19.3%) and 48 (54.5%) patients postoperatively, respectively. Age, sex, body mass index, comorbidities, previous endoscopic transsphenoidal surgery (ETS), preoperative hormone deficiency, preoperative copeptin level, GTR rate, and surgical complication rates were comparable among the three groups (Table 1). There was a higher proportion of craniopharyngioma (56.5%) and a lower proportion of pituitary adenoma (39.1%) in the permanent CDI group than in the non-CDI and transient CDI groups. Although tumor diameters were similar among the three groups, tumor height from planum sphenoidale in the permanent CDI group was substantially higher than that in the non-CDI group.

**Table 1:** Baseline characteristics of patients according to the presence of postoperative central diabetes insipidus.

Variables	Non-CDI (n = 48)	Transient CDI (n = 17)	Permanent CDI (n = 23)	*P*
Age, years	48.9 ± 16.7	43 ± 13.1	50.7 ± 10.2	0.233
Male	25 (52.1%)	4 (23.5%)	9 (39.1%)	0.112
BMI, kg/m^2^	25.7 (23.2, 27.9)	26.7 (21.8, 31.7)	26.5 (23.1, 28.8)	0.616
DM	6 (12.5%)	2 (11.8%)	1 (4.3%)	0.554
HTN	15 (29.2%)	3 (17.6%)	4 (17.4%)	0.442
Previous ETS	5 (10.4%)	0 (0%)	2 (8.7%)	0.625
**Sellar lesions**				
Craniopharyngioma	1 (2.1%)	1 (5.9%)	13 (56.5%)	** < 0.001**^**a,b**^
Meningioma	1 (2.1%)	0 (0.0%)	0 (0.0%)	0.656
Pituitary adenoma	46 (95.8%)	16 (94.9%)	9 (39.1%)	** < 0.001**^**a,b**^
Corticotroph	5 (10.4%)	3 (17.6%)	2 (8.7%)	0.647
Mammotroph	4 (8.3%)	1 (5.9%)	0 (0.0%)	0.365
Gonadotroph	15 (31.3%)	3 (17.6%)	1 (4.3%)	**0.033**^**a**^
Somatotroph	5 (10.4%)	0 (0.0%)	0 (0.0%)	0.110
Hormone inactive	17 (35.4%)	9 (52.9%)	5 (21.7%)	0.124
Rathke`s cleft cyst	0 (0.0%)	0 (0.0%)	1 (4.3%)	0.240
**Hormone deficiency**				
ACTH deficiency	0 (0.0%)	0 (0.0%)	0 (0.0%)	NA
TSH deficiency	0 (0.0%)	0 (0.0%)	0 (0.0%)	NA
FSH/LH deficiency	0 (0.0%)	1 (6.7%)	3 (9.5%)	0.154
Panhypopituitarism	0 (0.0%)	0 (0.0%)	0 (0.0%)	NA
Tumor diameter, mm	26.1 (21.5, 30.2)	29.2 (24.1, 40.6)	24.2 (21.0, 36.3)	0.341
Height from planum sphenoidale, mm	8.6 (5.1, 12.1)	11.8 (7.6, 17.2)	14.4 (10.3, 20.4)	**0.003**^**a**^
Preoperative copeptin, pmol/L	2.9 (2.1, 3.8)	3.1 (2.3, 3.9)	2.1 (1.6, 3.2)	0.139
Gross total resection	39 (81.3%)	13 (76.5%)	20 (87.0%)	0.689
**Postoperative complications**				
CNS Infection	0 (0.0%)	0 (0.0%)	1 (4.3%)	0.240
CSF Leakage	0 (0.0%)	0 (0.0%)	1 (4.3%)	0.240
CNS Hemorrhage	0 (0.0%)	0 (0.0%)	0 (0.0%)	NA
Hydrocephalus	0 (0.0%)	0 (0.0%)	1 (4.3%)	0.240

Data were represented as mean ± SD, number (proportion) or median (IQR). In bold *P*-values were calculated by one-way analysis of variance among the three groups. ^a^, *P* < 0.05 between non-CDI and permanent CDI; ^b^, *P* < 0.05 between transient CDI and permanent CDI; ^c^, *P* < 0.05 between non-CDI and transient CDI.

CDI, Central diabetes insipidus; BMI, Body mass index; Ex, ex-smoker or ex-drinker, respectively; DM, Diabetes mellitus; HTN, Hypertension; ETS, Endoscopic transsphenoidal surgery; NA, Not applicable; TSH, Thyroid stimulating hormone; ACTH, Adrenocorticotropic hormone; FSH, Follicular stimulating hormone; LH, Luteinizing hormone; Op., Operative; CNS, Central nervous system; CSF, Cerebrospinal fluid.

### Change between preoperative and 3 months postoperative copeptin levels

**Figure 1:**
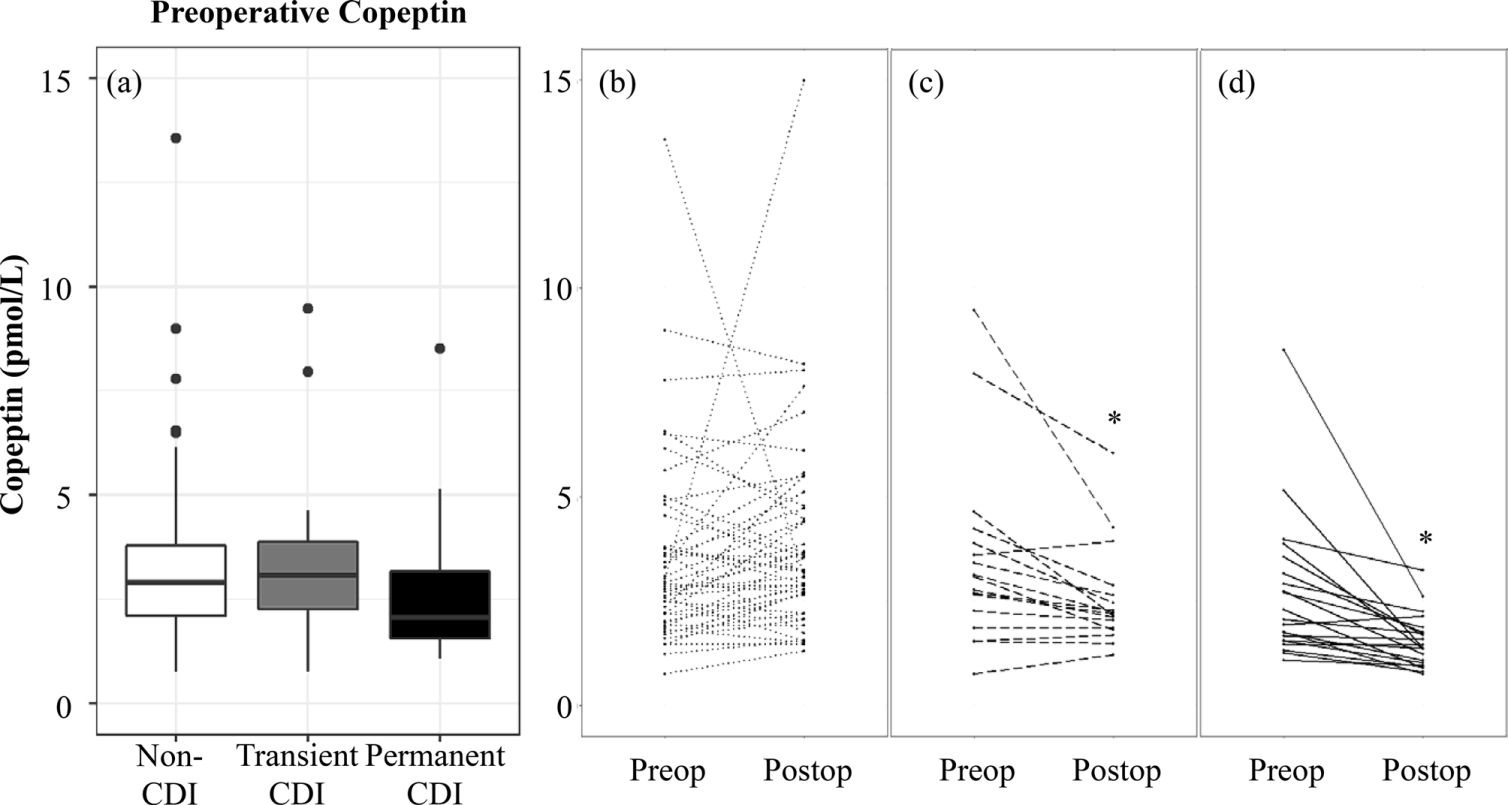
(**a**) Distributions of preoperative copeptin levels and change between preoperative and 3-month postoperative copeptin levels in subjects with (**b**) non-CDI, (**c**) transient and (**d**) permanent CDI. The figures show “Non-CDI,” “Transient CDI,” and “Permanent CDI” in the order from left to right. *P*-value versus Preop calculated using Wilcoxon signed-rank test was 0.130, 0.002, and < 0.001, respectively. * *P* < 0.05, versus Preop. Preop, Preoperative; Postop, Postoperative; CDI, Central diabetes insipidus.

Although preoperative copeptin levels of 12 patients in the permanent CDI group (57.1%) was < 2.6 pmol/L [18 in the non-CDI group (38.3%) and 5 in the transient CDI group (29.4%)], they showed no clinical signs of CDI and preoperative copeptin levels were comparable among three groups (Fig. 1a). Intriguingly, 3-month postoperative copeptin levels significantly declined from preoperative level in the permanent CDI group (*P* < 0.001, percentage difference =  − 42.2%), while there were no change of copeptin levels in the non-CDI group (*P* = 0.130, percentage difference = 9.6%). In addition, copeptin levels in the transient CDI group also significantly declined from preoperative levels (*P* = 0.002, percentage difference =  − 27.2%) (Fig. 1b–d).

### Diagnostic performance of 3-month postoperative copeptin level for permanent CDI

**Figure 2:**
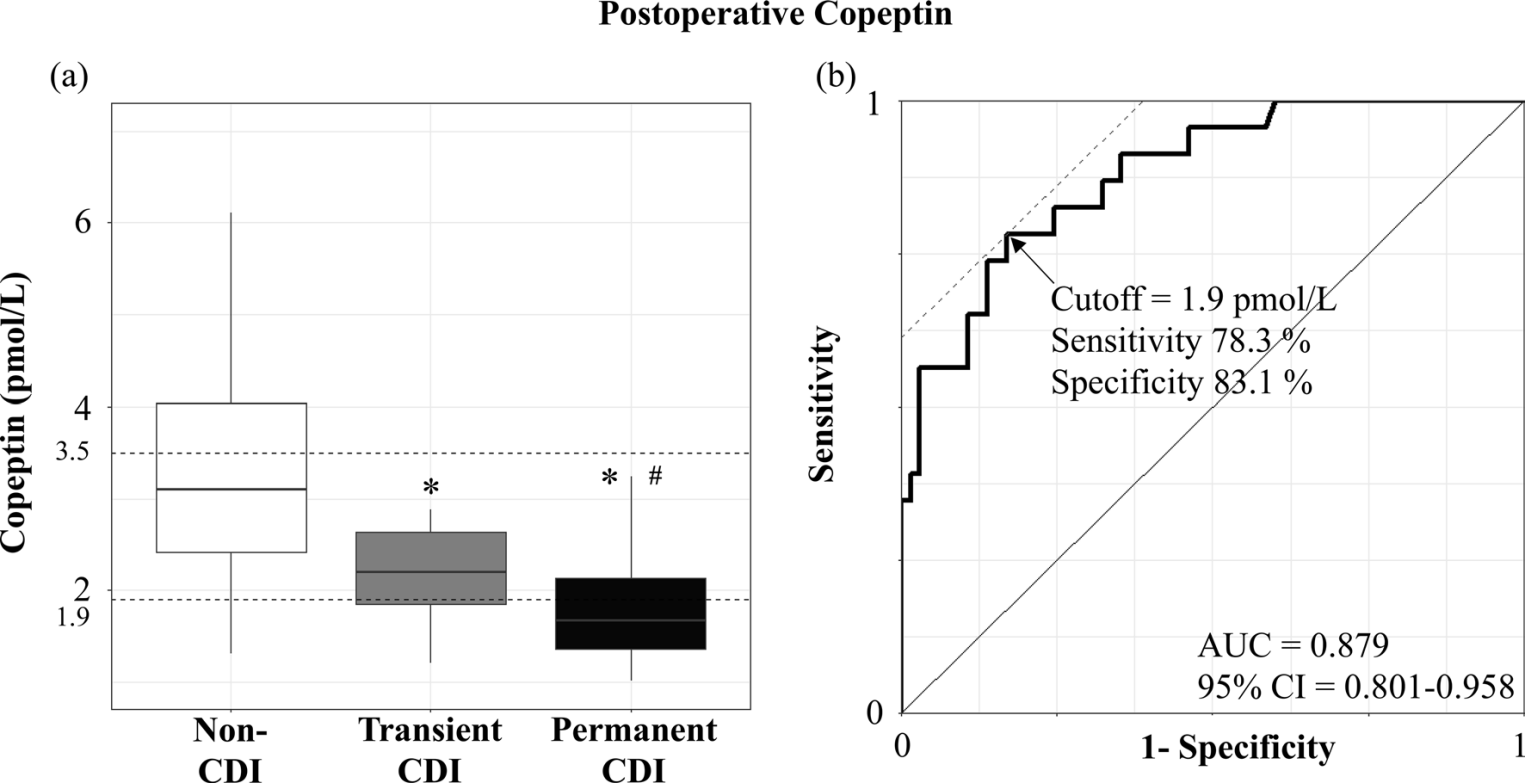
(**a**) Comparison of postoperative copeptin levels according to the presence of central diabetes insipidus at postoperative 3 months visit and (**b**) receiver operating characteristic curve analysis for diagnosing permanent central diabetes insipidus. (**a**) *P*-values versus non-CDI calculated using the Kruskal–Wallis with Dunn’s *post-hoc* analysis were 0.130, 0.002, and < 0.001, respectively. The dashed lines indicate the important cutoff values of 3.5 pmol/L and 1.9 pmol/L from the top to the bottom. Outlier values have been removed. *, *P* < 0.05, versus Non-CDI. #, *P* < 0.05 compared with Transient CDI. (**b**) Area under the curve (AUC) for postoperative copeptin levels was 0.879 (95% confidence interval = 0.801–0.958). The point indicated by the arrow is the cutoff point maximizing Youden index, which was 1.9 pmol/L. CDI, Central diabetes insipidus.

Given that copeptin level is strongly associated with serum sodium and osmolarity^18,24^, we compared serum postoperative sodium levels among the three groups. At 3 months postoperatively, all patients showed normal serum sodium levels. Furthermore, there were no significant differences in serum sodium levels among the three groups (median [IQR], 142.0 [141.0, 143.0] mEq/L in the non-CDI group; 143.0 [141.0, 144.0] mEq/L in the transient CDI group and 143.0 [142.0, 145.0] mEq/L in the permanent CDI group, *P* = 0.099) (data not shown). Under normal serum sodium levels, suggesting that all patients were treated properly, the 3-month postoperative copeptin level in the permanent CDI group was significantly lower than that in the non-CDI and transient CDI groups. The transient CDI group also showed lower 3-month postoperative copeptin levels than the non-CDI group [3.2 (2.7, 4.5) in the non-CDI group, 2.2 (1.9, 2.6) in the transient CDI group, and 1.4 (1.0, 1.8) in the permanent CDI group, *P* < 0.001] (Fig. 2a).

To investigate the diagnostic performance of 3-month postoperative copeptin level for permanent CDI, we performed ROC curve analysis (AUC = 0.879, 95% CI = 0.801–0.958) and revealed several cutoff values of the 3-month postoperative copeptin level for diagnosing permanent CDI (Fig. 2b). Three-month postoperative copeptin levels < 1.2 pmol/L maximized the specificity and positive predictive value (PPV) to 100%, with a sensitivity of 34.8% and a negative predictive value (NPV) of 81.3%. Using the Youden index, the optimal cutoff value of 3-month postoperative copeptin level was 1.9 pmol/L (sensitivity, 78.3%; specificity, 83.1%; PPV, 62.1%; NPV, 91.5%). The diagnostic performance of 1.9 pmol/L showed lower specificity and PPV with higher sensitivity and NPV than that of 2.6 pmol/L, as suggested by Bruno Allolio et al. for diagnosing CDI^19^. In addition, 3-month postoperative copeptin levels < 3.5 pmol/L maximized the sensitivity and NPV to 100% with a specificity of 40.0% and a PPV of 37.1%. (Table 2).

**Table 2:** Diagnostic performance of 3-month postoperative copeptin levels for central diabetes insipidus at postoperative visit.

Cut-off values, (n, %)	Sensitivity (%)	Specificity (%)	PPV (%)	NPV (%)	Accuracy (%)
< 1.2 pmol/L, (8, 9.1%)	34.8	100	100	81.3	83.0
< 1.9 pmol/L, (29, 33.0%)	78.3	83.1	62.1	91.5	81.8
< 2.6 pmol/L, (43, 48.9%)	87.0	64.6	46.5	93.3	70.4
< 3.5 pmol/L, (62, 70.5%)	100	40.0	37.1	100	55.7

Data in the Cut-off values column were represented as the cut-off of postoperative copeptin levels, the number and proportion of indicated patients.

PPV, Positive predictive value; NPV, Negative predictive value.

### Preoperative predictive factors for postoperative permanent CDI

**Figure 3:**
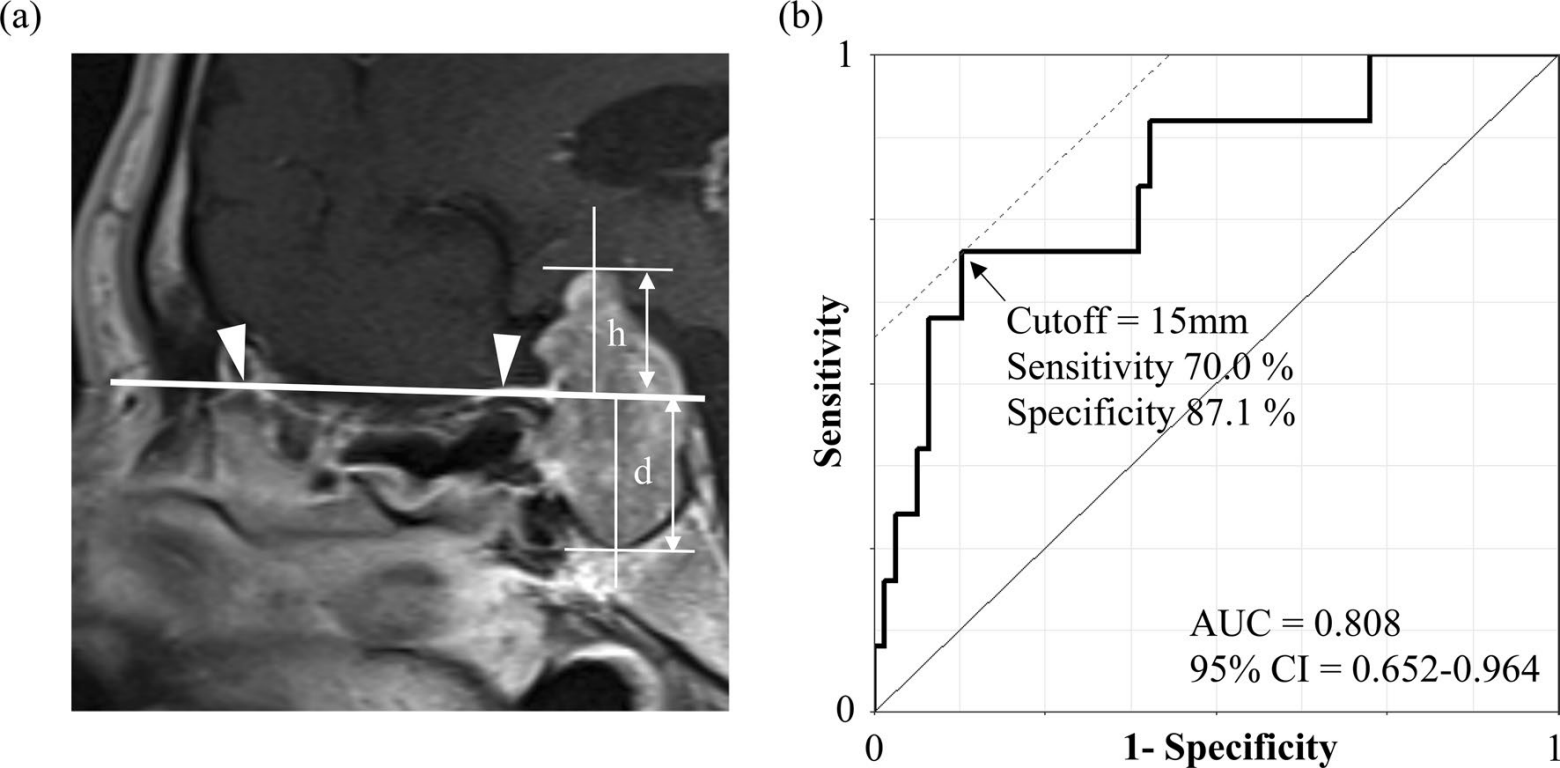
(**a**) Morphometric measurement of tumor height (h) and depth (d) from the planum sphenoidale and (**b**) receiver operating characteristic (ROC) curve analysis of tumor height from the planum sphenoidale for predicting permanent central diabetes insipidus. (**a**) Tumor height (h) is defined as the vertical distance from the conceptual line (white solid line) [drawn from the cristae galli (white arrowhead, left) to the planum sphenoidale (white arrowhead, right)] to the highest point of the tumor. (**b**) Area under the curve (AUC) for tumor height from the planum sphenoidale was 0.808 (95% confidence interval = 0.652–0.964). The point indicated by the arrow is the cutoff point maximizing the Youden index, which was 15 mm.

The presence of craniopharyngioma and tumor height from the planum sphenoidale were significantly different among the three groups. To determine the predictive performance of tumor height from the planum sphenoidale for postoperative permanent CDI, we performed ROC curve analysis with sellar lesions, except for craniopharyngiomas [the area under the curve (AUC) = 0.808, 95% CI = 0.652–0.964] and found that a tumor height of > 15 mm predicted permanent CDI with a sensitivity of 70.0% and specificity of 87.1% (Fig. 3a,b).

Logistic regression analysis revealed the risk factors predicting postoperative permanent CDI (Table 3). In a univariate model, craniopharyngioma and a tumor height of > 15 mm were strongly associated with postoperative CDI, and the significance remained in a multivariate model (craniopharyngioma: OR = 283.3, 95% CI = 27.7–8349, *P* < 0.001; tumor height: OR = 29.3, 95% CI = 5.2–280, *P* < 0.001). In addition, a stepwise backward selection procedure indicated that craniopharyngioma and high tumor height > 15 mm were significantly interacted with each other. These results were consistent with previous reports^13,14^.

**Table 3:** Preoperative prediction models for postoperative permanent diabetes insipidus.

Variables	Univariate model		Multivariate model	
	OR (95% CI)	*P*	OR (95% CI)	*P*
Craniopharyngioma	34.1 (7.8, 243.4)	< 0.001	141.3 (18.4, 3150.3)	< 0.001
Height from planum sphenoidale > 15 mm	4.9 (1.7, 15.0)	0.004	13.7 (3.2, 73.8)	< 0.001
Tumor diameter	1.0 (1.0, 1.1)	0.367	–	–
Gross total resection	1.4 (0.4, 6.6)	0.643	–	–
Postoperative complications	NA	0.991	–	–

OR, Odds ratio; CI, Confidence Interval; NA, Not applicable.

## Discussion

We demonstrated that 3-month postoperative copeptin levels significantly decreased compared with preoperative copeptin levels in the transient and permanent CDI group. Furthermore, both the permanent and transient CDI groups showed lower serum copeptin levels than the non-CDI group at the 3-month postoperative visit. Finally, a 3-month postoperative copeptin level of 1.9 pmol/L was identified as the diagnostic cutoff value for permanent CDI, and ≥ 3.5 pmol/L excluded permanent CDI. In addition, we found that craniopharyngioma and tumor height from the planum sphenoidale > 15 mm were independently associated with the incidence of postoperative permanent CDI.

Three previous studies using immediate postoperative copeptin levels showed time-dependent changes in copeptin and the association between the loss of stress-induced copeptin peak and the occurrence of postoperative CDI^21–23^. The low immediate postoperative copeptin levels implicated postoperative impairment of posterior pituitary gland function under stress-induced state and predicted the occurrence of postoperative CDI. Due to the lack of immediate postoperative blood samples, we could not analyze the immediate postoperative copeptin level. Instead, in the present study, both preoperative and 3-month postoperative copeptin levels in each patient were analyzed. Notably, 3-month postoperative copeptin levels significantly decreased from preoperative levels in the transient CDI group, although they were no longer taking desmopressin at 3 months postoperatively. Given that complete CDI occurs when AVP secretion is significantly reduced, AVP secretion of patients with postoperative transient CDI is likely to be below the extent of complete CDI within several days postoperatively. The result of this study suggested that AVP secretion of the transient CDI group is recovered soon after, but still lower than that of the non-CDI group at postoperative 3 months visit. Collectively, since the copeptin level reflects the posterior pituitary function^25^, the posterior pituitary reserve of the transient CDI group may be attenuated even if desmopressin is not needed.

A previous study reported that a single baseline copeptin level < 2.6 pmol/L after 8 h of water deprivation showed the optimal diagnostic performance to discriminate complete CDI from primary polydipsia^19,20^. We investigated several cutoff values of 3-month postoperative copeptin levels with variable diagnostic performances for postoperative permanent CDI. We suggest that the cutoff value of 1.9 pmol/L results in better accuracy than that of the 2.6 pmol/L. However, the results were not comparable due to several differences between the two studies.

We retrieved the cutoff value for permanent CDI, including asymptomatic patients, whereas previous studies analyzed in patients with polyuria-polydipsia syndrome. We did not measure serum copeptin levels after the standardized test, such as water deprivation or hypertonic saline test, although we collected blood samples in a fasting state but except for water intake. In detail, patients were instructed not to eat any food at least 12 h before blood sampling and to take a desmopressin of hours of sleep as scheduled and not to take a morning dose of desmopressin, but water intake was not abstained. Since the antidiuretic effect of desmopressin acetate lasts up to 8 h, and the half-life of it is 1.1 h, we expected that serum copeptin level would not be influenced by desmopressin acetate in this study. Despite our expectations, taking desmopressin acetate and water intake could reduce serum copeptin levels. In addition, the assays for measuring serum copeptin were different in that we used time-resolved amplified cryptate emission (TRACE) technology, while Fenske et al. used a one-step assay with coated-tube technology. Although the copeptin levels measured by each method were comparable^26^, each method has different minimal detection limits and there are no international standardized calibration references. These reasons could possibly explain the discrepant copeptin cutoffs.

In addition, we confirmed that under normal serum sodium, all patients with 3-month postoperative copeptin level of < 1.2 pmol/L developed permanent CDI, whereas no patients with 3-month postoperative copeptin level of ≥ 3.5 pmol/L did not experience permanent CDI. The cutoff value of 3.5 pmol/L was also suggested as the value of the highest accuracy after arginine-stimulated copeptin measurement^5^. The 3-month postoperative copeptin level between 1.2 and 3.5 pmol/L may require further confirmation tests such as hypertonic saline test or arginine-stimulation test.

Previously identified risk factors for post-ETS CDI were mostly limited to clinical factors. As craniopharyngioma and Rathke’s cleft cysts are usually located either in or above the sella turcica^13^, postoperative CDI is unavoidable if complete resection is attempted. In pituitary adenomas, visual abnormalities, suprasellar extension, large tumor diameter, extensive surgical resection, and intraoperative CSF leak were associated with an increased incidence of permanent CDI^8,10–12^. As the extent of surgical manipulation and risk of intraoperative CSF leak was inevitably determined by the extent of the tumor, attention has been focused on tumor size^6,8,12,15^. Previous studies have used the tumor's longest diameter or cephalocaudal diameter without any baseline^14^ as a parameter. We specified tumor height describing the suprasellar extension of the tumor based on the imaginary line from the cristae galli to the planum sphenoidale in the midsagittal view. This method standardized the shift distance of the pituitary gland and stalk by tumor from the unknown previous normal sellar fossa and avoids the bias from the relatively higher height of the tumor due to sellar enlargement with tumor growth. We, in the end, revealed that the tumor height from the planum sphenoidale to > 15 mm was a significant risk factor for postoperative permanent CDI instead of the longest diameter of the tumor.

The main strength of the present study is that we measured the paired samples preoperatively and postoperatively at 3 months' visit in each patient. Thus, we observed the postoperative diminished reserve of the posterior pituitary gland through change between preoperative and 3 months postoperative copeptin levels in both the transient and permanent CDI groups. In addition, using serum copeptin levels at the postoperative 3 months visit, we identified the diagnostic cutoff value of copeptin for postoperative CDI at the stable state under a normal range of serum osmolarity and sodium.

Nonetheless, the limitations of the study should be mentioned. Our study was retrospective in nature. Data were retrieved from medical records, and potential bias or confounders were inevitable. Using the stored serum samples preoperatively and 3-month postoperatively, we could not analyze the copeptin level after the osmotic stimulation test or immediate postoperative copeptin level. In addition, we used serum samples for the copeptin assay stored at − 80 °C for 2.4 ± 0.4 years. The storage may lead to decreased copeptin levels and result in the lower cutoff values in this study, and AVP could not be assayed simultaneously owing to its peptide instability. Making an appropriate diagnosis of postoperative CDI in an outpatient clinic is difficult due to limited information. History taking can not distinguish polyuria from frequenturia, and there have been various causes resulting in polyuria-polydipsia symptoms^27,28^. Based on our result, we expect that the copeptin levels in a fasting state provide clinicians additional evidence for the reservoir of the posterior pituitary gland with a simple biochemical test.

In this study, we suggest that simultaneous preoperative and postoperative serum copeptin measurements provide valuable information for investigating the changes of posterior pituitary function after transsphenoidal surgery. Three-month postoperative copeptin levels under normal serum sodium levels may also be useful for diagnosing postoperative permanent CDI. Under normal sodium levels, 3-month postoperative copeptin levels below 1.9 pmol/L may diagnose permanent CDI, while those above 3.5 pmol/L may exclude permanent CDI. For 3-month postoperative copeptin levels to be used as a robust diagnostic for permanent CDI, a few caveats should be addressed in the prospective study such as the relationship between immediate postoperative copeptin levels and 3-months postoperative copeptin levels, the effect of desmopressin intake on serum copeptin levels, and the international standardized copeptin measuring protocol. Collectively, copeptin measurement would provide clinicians the opportunity to assess posterior pituitary function at postoperative visits and enable effective interventions to manage permanent CDI.

## Materials and methods

### Ethics and approval

This study was approved and the requirement for written informed consent was waived by the Institutional Review Board of Seoul National University Hospital (No. 2006–169-1136). This study was performed according to the Declaration of Helsinki.

### Patients

This retrospective observational study was conducted as a part of the Seoul National University Pituitary Disease Cohort Study (SNU-PIT) (ClinicalTrial.gov, NCT03474601), a prospective cohort study of patients with pituitary diseases at Seoul National University Hospital. We included 88 patients (aged ≥ 18 years) who underwent ETS due to pituitary gland or stalk lesions from August 2017 to October 2018. We collected the clinical data and blood samples preoperatively and 3-month postoperatively. Patients with preoperative CDI and those who failed to follow-up for 3 months after surgery were excluded.

### Diagnosis of postoperative CDI

The diagnosis and management of postoperative CDI followed a protocol of our previous study and were similarly described here^14^. CDI was diagnosed when patients had a large urine output (> 50 mL/kg/24 h) with either a high serum sodium concentration (> 145 mmol/L) or a large increase (≥ 3 mmol/L) in serum sodium concentration between two consecutive tests after surgery. Once CDI was diagnosed during admission, an intravenous crystalloid solution was administered to maintain fluid balance, and 1 μg intravenous desmopressin was injected intermittently with follow-up urine output and laboratory findings (urine specific gravity, urine osmolarity, serum osmolarity, and serum sodium concentration). If CDI did not recover until discharge, patients were prescribed oral desmopressin. Patients with definite intraoperative stalk injury were instructed to take 0.1 mg oral desmopressin at 12-h intervals and stop taking it when their urine output decreased below 50 mL/kg/24 h, whereas those without definite intraoperative stalk injury were instructed to take 0.1 mg oral desmopressin when their urine output increased above 50 mL/kg/24 h.

CDI included both transient and permanent DI, which were divided according to whether or not desmopressin was prescribed for > 3 months after surgery^10,29^.

### Data collection

Age, sex, height, weight, social history, preoperative comorbidities, previous treatments (trans-sphenoidal surgery, craniotomy, gamma knife surgery, and radiotherapy), and pathological results were evaluated. Diabetes mellitus and hypertension were defined by laboratory data (hemoglobin A1c ≥ 6.5% for diabetes mellitus), medical history, or pharmacological treatment.

Gross total resection (GTR) was defined as the absence of a visible tumor in the surgical view and immediate postoperative magnetic resonance images taken within 24 h. Central nervous system (CNS) infection was diagnosed based on the Center for Disease Control and Prevention Criteria^30^. The presence of postoperative cerebrospinal fluid (CSF) leakage was assessed using the Kelly grading system^31^, and grades 1–3 were diagnosed as positive for CSF leakage. Postoperative CNS hemorrhage and hydrocephalus were assessed using immediate and follow-up postoperative brain images.

Tumor height was measured on preoperative magnetic resonance images. Based on the imaginary line from the cristae galli to the planum sphenoidale at the midsagittal view, tumor diameter was measured as the distance vertically to the line from the highest point to the lowest point^14^. Tumor height was measured as the distance from the line to the highest point in the vertical direction (Fig. 3a).

Anterior pituitary function was assessed preoperatively in all patients. The results were interpreted according to our previous study protocol^14,32^, which were described similarly here. Adrenocorticotropic hormone (ACTH) deficiency was defined as peak serum cortisol concentration below 18 µg/dL in a short Synacthen test with a low to normal serum ACTH concentration. Thyroid-stimulating hormone (TSH) deficiency was defined as a serum-free thyroxin concentration below 0.7 ng/dL with a low to normal serum TSH concentration. Gonadotropin deficiency in male patients was defined as early morning serum testosterone concentration below 2.7 ng/mL with a low to normal serum follicle-stimulating hormone (FSH) and luteinizing hormone (LH)]. Gonadotropin deficiency in premenopausal female patients was defined as the absence of menstruation without elevated FSH and LH levels, while that in postmenopausal female patients was defined as low to the normal serum concentration of FSH and LH.

### Surgery

All patients underwent ETS, and details are described in our previous report^33^. In pituitary adenomas, the tumor was dissected from the compressed pituitary gland in a fashion of extracapsular dissection, and a gentle dissection with microdissectors and cottoid pledges were performed in patients without pseudocapsules. Microcurettage was not used in any case to prevent damage to the thin pituitary gland. In craniopharyngioma, the pia over the pituitary stalk was dissected to reveal the dissection plane between the tumor and pituitary stalk, following the dissection of the superior hypophyseal artery. Microsurgical dissection techniques were adopted to dissect the tumor from the pituitary gland, stalk, and hypothalamus, and blunt dissection was not performed. After tumor resection, vasodilator-soaked pledgets were applied to the bilateral superior hypophyseal arteries to prevent vasospasm by surgical manipulations.

### Copeptin measurement

Blood samples were collected from patients preoperatively and 3 months after surgery in a fasting state, which indicate that patients were instructed not to eat any food at least for 12 h with free access to water, to take a desmopressin of hours of sleep as scheduled and not to take a morning dose of desmopressin. Blood from the serum-separating tube was centrifuged at 4000 rpm for 5 min, and a serum aliquot was immediately frozen and stored at − 80 °C until analysis. Serum samples were obtained from the Seoul National University Hospital Biobank, a member of the National Biobank Korea. All samples were thawed once and analyzed according to the manufacturer’s recommendations described below in detail.

Copeptin levels were measured by TRACE using a commercial sandwich immunoluminometric assay, the BRAHMS Copeptin KRYPTOR kit on a BRAHMS KRYPTOR compact plus analyzer (BRAHMS GmbH, Hennigsdorf, Germany) as previously described^2,24^, which could measure copeptin levels 0.7–500 pmol/L. According to the manufacturer’s recommendation, precision of the copeptin assay was tested at two levels (5.21 pmol/L and 97.10 pmol/L) by running five replicates for each run for five days. Within-run and between-run precision coefficients of variations were 6.0% and 2.2%, respectively, which showed stable results. The analytical measurable range was 1.25–106 pmol/L. In general, ± 50% for the low concentration range and ± 10% for the high concentration range are used as the actual applicable range (permissible range). Thus, the final analytical measureable range can be applied up to 0.63–117 pmol/L.

There was no cross-reactivity between copeptin and AVP due to considerably different amino acid sequences.

### Statistical analysis

Categorical variables were presented as the number (proportion). For continuous variables, the normalities of the data distribution were assessed using the Shapiro–Wilk test. According to the *P*-values calculated from the Shapiro–Wilk test, continuous variables were differently presented as means ± standard deviations or medians (interquartile ranges [IQR]). For categorical variables, *P*-values were calculated using the chi-square test and the false discovery method was used for *post-hoc* analysis. For continuous variables, *P*-values were calculated by one-way analysis of variance (ANOVA) with Tukey’s *post-hoc* analysis if the variables met the ANOVA assumption of normality, while *P*-values were calculated using the Kruskal–Wallis test with Dunn’s *post-hoc* analysis if the variables did not meet the ANOVA assumption of normality. For paired continuous variables, *P*-values were calculated using the Wilcoxon signed-rank test because the measured copeptin levels were not normally distributed.

Receiver operating characteristic (ROC) curve analysis was performed to evaluate the discriminative power of the significant variables. The optimal cutoff value of height from planum sphenoidale was set to a value maximizing the Youden index (sensitivity + specificity − 1). To identify predictors of postoperative CDI, variables which have reported be associated to CDI were analyzed with univariate logistic regression analysis and those with *P*-values < 0.1 in the univariate model were included in the multivariate logistic regression analysis. To assess whether associations between variables, interaction terms were included in multivariate logistic regression, and a reduced model was selected using a stepwise backward selection procedure. To set variable cutoffs of postoperative copeptin level and quantify their diagnostic performance, we used R package ‘OptimalCutpoints’^34^. A *P*-value < 0.05 was considered statistically significant. All statistical analyses were performed using R Statistical Software (version 4.0.3).

## Consent for publication

All authors agreed for publication of the manuscript.

## Data Availability

All custom code to analyze data and generate figures in this study are available from the corresponding author upon reasonable request.
